# Biosorption of Cr(VI) by immobilized waste biomass from polyglutamic acid production

**DOI:** 10.1038/s41598-020-60729-5

**Published:** 2020-02-28

**Authors:** Chao Zhang, Hui-Xue Ren, Chuan-Qing Zhong, Daoji Wu

**Affiliations:** 1 School of Municipal and Environmental Engineering, Shandong Jianzhu University, JiNan, 250101 China; 2 Co-Innovation Center of Green Building, JiNan, 250101 China

**Keywords:** Environmental biotechnology, Industrial microbiology

## Abstract

Waste biomass from γ-polyglutamic acid production was used as an adsorbent to remove Cr(VI) from wastewater. Waste biomass was entrapped in sodium alginate to enhance performance. Orthogonal array design was used to optimize biosorption of Cr(VI) by immobilized waste biomass. The optimal adsorption conditions for immobilized waste biomass were as follows: pH 7.0, initial Cr(VI) concentration of 200 mg/L, 35 °C, waste biomass of 2 g/L, 60 min. Under these conditions, the absorption efficiency of Cr(VI) was 96.38 ± 0.45%. When the waste biomass was treated with 1 mol/L HCl for 1 h, the desorption rate could reach 94.42 ± 0.87%. It was shown that the adsorption kinetics followed the Freundlich adsorption model, indicating that the adsorption of Cr(VI) by bacteria was mainly based on multi-molecular layer adsorption. The absorption conditions of waste biomass were mild (pH 6.0–7.5, 20–35 °C) and easily operated. These investigations lay a foundation for reducing the pollution of γ-polyglutamic acid production, turning the biomass waste into a useful adsorbent for wastewater treatment.

## Introduction

Chromium (Cr) is a prominent metal pollution in modern industrialized world as a result of the extensive application of this metal in a variety of industries including metallurgy, mining, electroplating, printing and dyeing industries^1^. Wastewater discharged from these industries contain a significant amount of the hexavalent ionic form of this metal, which has attracted wide attention due to its toxicity, high carcinogenicity and mutagenicity^2^. Therefore, the effective treatment of wastewater containing Cr(VI) has become one of the hot issues in environmental sciences nowadays. Today, the most common methods for treating wastewater containing heavy metal ions are chemical precipitation, membrane filtration, liquid phase extraction, biosorption and ion exchange^3^. Among them, adsorption is the most promising method in the treatment of heavy metals, which already showed potentials in a many practices^4^. In particular, biosorption has attracted wide attention because of its low cost, easy operation and high removal rate^5^.

Gamma-polyglutamic acid (γ-PGA) is a water-soluble macromolecule material synthesized by microbial fermentation. It is synthesized by the polymerization of L-glutamic acid and D-glutamic acid via gamma-amide bonds^6^. Because of its reduced pollution to the environment, excellent biodegradability, film forming property, fibrogenicity, water-holding capacity and other special physical and chemical properties, it has now been widely used in medicine, agriculture, environmental protection, cosmetics, food and other industries^7–10^. With the increase of the demand and production of γ-PGA each year, a large number of waste biomass is produced, which unfortunately is another source of pollution if discharge directly without proper processing^11^.

Traditional methods for the treatment of Cr(VI) containing wastewater are expensive. In contrast, waste biomass is a byproduct of γ-PGA industry, and can drastically reduce treatment costs if it finds applications in the treatment of heavy metal-containing wastewater. *Bacillus subtilis* is the main producing strain of γ-PGA. Previous studies have reported the use of *B. subtilis* as an adsorbent. For example, Sukumar *et al*.^12^ reported the Cr(VI) removal using *B. subtilis* SS-1 isolated from soil samples of the electroplating industry. The removal efficiency was found to be 98.7% at 100 mg/L initial Cr(VI) concentration, pH 2 and 0.1 g/L biosorbent. Sivaprakash *et al*.^13^ reported that the *B. subtilis* biomass has the maximum biosorption rate of 48.64% at 100 mg/L initial chromium concentration, pH 2, 30 °C and 2 g/L biomass. The above-mentioned literature showed that *B. subtilis* had a strong adsorption capacity for Cr(VI). Therefore, the *B. subtilis*-containing waste biomass from γ-polyglutamic acid production has the potential to absorb Cr(VI) from wastewater, which was investigated in this study. The use of waste biomass to remove Cr(VI) from wastewater conforms with the concept of “treating wastewater with waste”. This concept has been applied by many scientists for the removal of Cr(VI). For example, Rossi *et al*.^14^ reported the removal of Cr(VI) using chemical and thermal treated discarded *Saccharomyces cerevisiae* at an efficiency of 99.66%. Mona *et al*.^15^ reported the removal of Cr(VI) by spent cyanobacterial biomass from a hydrogen fermenter at an efficiency of 80–90%. However, there is no report on the removal of Cr(VI) by waste biomass from γ-PGA production. The use of immobilized waste biomass in this work has an additional advantage in comparison with previous work: microbial cells are small, and are difficult to separate from aqueous solution, causing secondary pollution; while immobilizing cells can solve this problem and further lead to higher efficiency, stronger stability and better solid-liquid separation effect.

In this study, the adsorption of Cr(VI) in wastewater was performed using immobilized waste γ-PGA biomass as adsorbent. The adsorption conditions of waste γ-PGA biomass were optimized by orthogonal array design. The desorption conditions, adsorption isotherm and adsorption kinetics were studied in order to provide reference for the practical treatment of Cr(VI) in wastewater.

## Results

### Effect of time on adsorption rate

**Figure 1:**
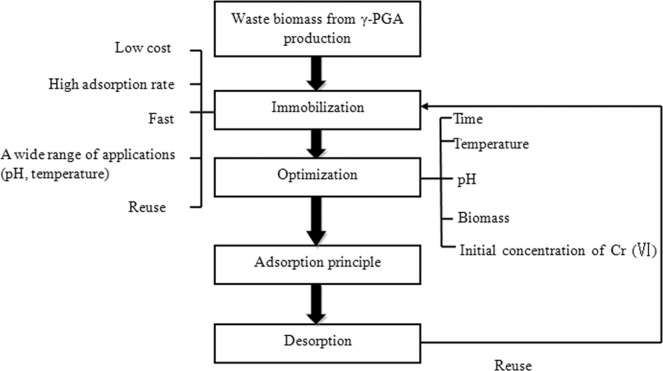
Flow chart.

**Figure 2:**
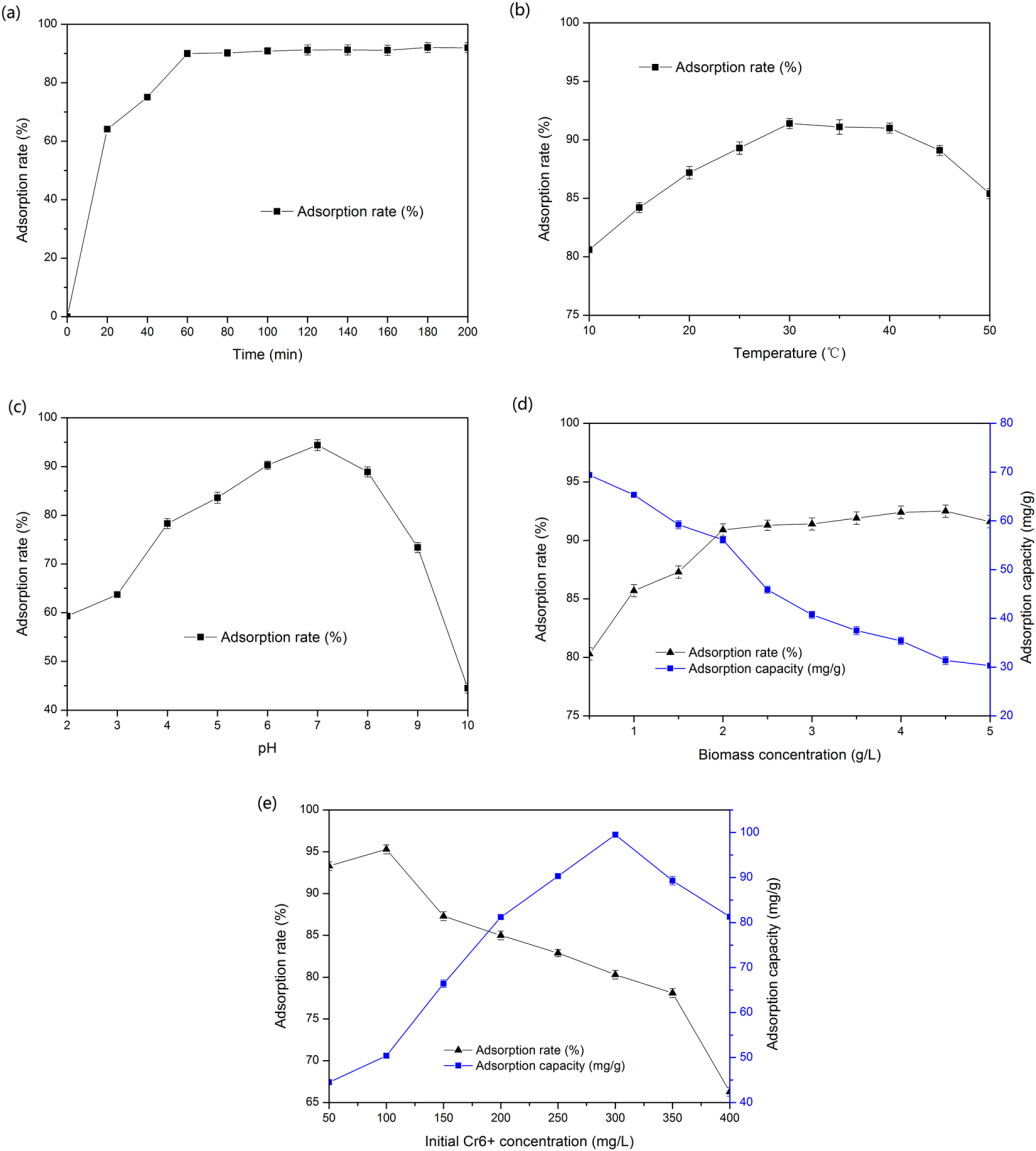
Effect of time (**a**), temperature (**b**), pH (**c**), biomass concentration (**d**) and initial concentration of Cr(VI) (**e**).

The process diagram of the whole experiment is shown in Fig. 1.The effect of adsorption time on the adsorption rate was investigated in reactions with the following parameters: temperature 25 °C, initial Cr(VI) concentration 50 mg/L, 3 g/L biomass pellets, pH 5, shaking speed 150 rpm. The adsorption results are shown in Fig. 2(a). It was shown that the adsorption rate of Cr(VI) to the immobilized biomass increased rapidly with time in the first 20 min. The adsorption rate reached 90.01 ± 1.13% at 60 min, and then increases slowly with time, reaching equilibrium at 180 min, when the adsorption rate reaches 92.06 ± 0.97%. Therefore, the adsorption time was set at 60 min in further optimization of Cr(IV) adsorption experiments.

### Effect of temperature on adsorption rate

The effect of temperature on the adsorption rate was investigated. The results are shown in Fig. 2(b). It can be seen that the adsorption rate maintained at a high level at 30–40 °C, and reached maximum of 91.01 ± 0.44% at 30 °C. When the temperature exceeded 30 °C, the adsorption rate decreased with the increase of temperature.

### Effect of pH on adsorption rate

From Fig. 2(c), it can be seen that the adsorption rate of waste biomass for Cr(VI) increases significantly with the increase of pH value when the pH value is 2–7. The maximum adsorption rate of 94.40 ± 0.83% was reached at pH 7. It is suspected that when the pH is low, the functional groups on the cell wall are protonated, while when the pH increased, H^+^ would dissociated from the functional groups, exposing more negative charged groups, which was beneficial to the binding of metal ions. However, too high pH would cause metal ions to form hydroxide precipitation, sabotaging the adsorption process^16^. Therefore, the optimum pH for the adsorption of Cr(VI) by waste biomass was 7.

### Effect of waste biomass concentration on adsorption rate

Figure 2(d) shows that the biomass content is positively correlated with the removal rate of Cr(VI), but negatively correlated with the adsorption capacity. The reason might be that when the amount of metal ions in the solution was fixed, the smaller the concentration of the adsorbent was, higher number metal ions were surrounded around it, higher concentrations of the adsorbent could combine with metal ions to an larger extent, leading, to greater adsorption amount of the adsorbent per unit mass^17^. With these considerations, the dosage of bacteria was set at 2 g/L.

### Effect of initial Cr(VI) concentration on adsorption rate

Figure 2(e) shows that with the increase of initial concentrations of Cr(VI) in solution, the adsorption rate of Cr(VI) gradually decreases, while the adsorption capacity first increases and then decreases. This might be because the number of active sites adsorbed by immobilized biomass was limited. When the initial concentration of Cr(VI) in solution was 100 mg/L, the number of active sites adsorbed by immobilized biomass was excessive in relative to Cr(VI). At this time, the adsorption rate of Cr(VI) was the highest (95.3 ± 0.72%) and the adsorption capacity was low (50.4 ± 0.32 mg/g). With the increase of initial concentration of Cr(VI) in solution, the adsorption sites on the surface of biomass become relatively smaller. Therefore, the adsorption rate decreases and the adsorption capacity increases. When the number of Cr(VI) in solution is equal to the adsorption sites on the surface of biomass, the adsorption capacity reaches saturation, and then the maximum adsorption capacity is 99.5 ± 0.52 mg/g. Thereafter, the adsorption capacity decreases with the increase of initial concentration of Cr(VI) in solution.

### Adsorption optimization by orthogonal experiment

An orthogonal experiment was performed to optimize the combination of various parameters. Five factors at four levels, time (40, 50, 60, 70 min), temperature (20, 25, 30, 35 °C), pH (6, 6.5, 7, 7.5), biomass concentration (1.5, 2, 2.5, 3%) and initial concentration of Cr(VI) (200, 250, 300, 350 mg/L), were selected for optimization (Table 1).

**Table 1:** Orthogonal experiment design and results.

Trial no.	A Time(min)	B T ( °C)	C pH	D Biomass concentration (g/L)	E Cr (VI)concentration(mg/L)	Adsorption rate (%)
1	40	20	6.0	1.5	200	71.81 ± 0.42
2	40	25	6.5	2	250	74.22 ± 0.32
3	40	30	7.0	2.5	300	77.18 ± 0.31
4	40	35	7.5	3	350	75.21 ± 0.33
5	50	20	6.5	2.5	350	85.10 ± 0.36
6	50	25	6.0	3	300	84.12 ± 0.38
7	50	30	7.5	1.5	250	83.14 ± 0.32
8	50	35	7.0	2	200	93.01 ± 0.30
9	60	20	7.0	3	250	89.06 ± 0.39
10	60	25	7.5	2.5	200	91.04 ± 0.33
11	60	30	6.0	2	350	95.03 ± 0.39
12	60	35	6.5	1.5	300	93.02 ± 0.33
13	70	20	7.5	2	300	83.11 ± 0.31
14	70	25	7.0	1.5	350	80.16 ± 0.36
15	70	30	6.5	3	200	82.14 ± 0.42
16	70	35	6.0	2.5	250	84.12 ± 0.34
k_1_	74.605	82.270	83.770	82.032	84.500	
k_2_	86.343	82.385	83.620	86.343	82.635	
k_3_	92.037	84.373	84.852	84.360	84.358	
k_4_	82.382	86.340	83.125	82.632	83.875	
R	17.432	4.070	1.727	4.311	1.865	

Adsorption results indicated that the influential order of the five factors on the adsorption rate was A > D > B > E > C (Table 1). According to variance analysis, the time (p < 0.05) for the adsorption rate was significant, whereas B, C, D and E were not significant factors (Table 2). It can be see from Table 1 that k_3_ is time, k_4_ is the largest in temperature, k_3_ is the largest in pH, k_2_ is the largest in biomass concentration, and k_1_ is the largest in initial concentration of Cr(VI). Therefore, the optimal adsorption condition was deduced as: biomass 2 g/L, 60 min, 35 °C, initial Cr(VI) concentration of 200 mg/L and pH 7.0. In order to verify the optimal condition, adsorption under this condition was studied, and the adsorption rate reached 96.38 ± 0.45%. Therefore, this deduced condition was proved to be the best combination of different parameters.

**Table 2:** Variance analysis of orthogonal experiment.

Factors	SS	df	F	F_0.05_	Significant
A	643.484	3	101.193	9.280	*
B	44.462	3	6.992	9.280	
C	6.359	3	1.000	9.280	
D	45.032	3	7.082	9.280	
E	8.627	3	1.357	9.280	
Error	6.36	3			

### Adsorption kinetics analysis

Adsorption kinetics is mainly used to analyze the relationship between adsorption rate and adsorption time. Adsorption rate controls the time needed to reach adsorption equilibrium. It is an important parameter to measure adsorption. In the study of adsorption kinetics, Lagergren quasi-first-order kinetic model and Lagergren quasi-second-order kinetics model are usually used to describe the relationship between the mass concentration of metal ions and time. The quasi-first-order kinetic model is based on the assumption that diffusion is controlled by adsorption. The quasi-second-order kinetics model is based on the assumption that the adsorption rate is controlled by chemical adsorption process. The linear forms of quasi-first-order kinetic model and second-order kinetics model are as follows^18^:

1 $$\mathrm{ln}\,({{\rm{q}}}_{{\rm{e}}}-{{\rm{q}}}_{{\rm{t}}})={{\rm{lnq}}}_{{\rm{e}}}-{{\rm{k}}}_{1}{\rm{t}}$$

2 $$\frac{{\rm{t}}}{{{\rm{q}}}_{{\rm{t}}}}=\frac{1}{{{\rm{k}}}_{2}{{\rm{q}}}_{{\rm{e}}}^{2}}+\frac{{\rm{t}}}{{{\rm{q}}}_{{\rm{e}}}}$$

where k_1_ was pseudo-first order rate constant (h^−1^), k_2_ was quasi-second-order rate constant (g·mg^−1^·h^−1^), t was adsorption time (h), q_t_ was adsorption amount at t time (mg·g^−1^), q_e_ was adsorption amount at equilibrium (mg·g^−1^).

According to the parameters of Table 3, the fitting effect of quasi-second-order kinetic equation was better and R^2^ is higher, which indicated that quasi-second-order kinetic model could describe the adsorption process of waste biomass to Cr(VI) more accurately. According to the mechanism of quasi-second-order kinetic equation, it could be inferred that physical diffusion and chemical adsorption coexist in the process of adsorption of Cr(VI) by waste biomass. Chemical adsorption was dominant, and the effect of physical diffusion on the rate of chemical adsorption could be neglected.

**Table 3:** The fitting parameters of quasi-first-order and quasi-second-order reaction kinetic equations.

Quasi-first-order kinetic model		Quasi-second-order kinetics model	
k_1_/ h^−1^	R^2^	k_2_/(g·mg^−1^·h^−1^)	R^2^
1.3712	0.9611	1.0302	0.9992

### Adsorption isotherm

Under the optimal adsorption conditions, the adsorption isotherm of chromium on bacteria was studied. The isothermal adsorption model can be used to describe the affinity between adsorbate and adsorbent and the surface adsorption characteristics^19^.

Langmuir adsorption model:

3 $$\frac{1}{{{\rm{q}}}_{{\rm{e}}}}=\frac{1}{{{\rm{abc}}}_{{\rm{e}}}}+\frac{1}{{\rm{a}}}$$

Freundlich adsorption model:

4 $${{\rm{lgq}}}_{{\rm{e}}}={\rm{lgK}}+\frac{1}{{\rm{n}}}{{\rm{lgc}}}_{{\rm{e}}}$$

where q_e_ was the equilibrium adsorption capacity (mg/g), c_e_ was the equilibrium concentration of Cr(VI) in solution (mg/L), a and b were Langmuir adsorption constants, K and n were Freundlich adsorption constants.

Langmuir and Freundlich isothermal adsorption models are used to fit the experimental data^20^. The adsorption parameters are shown in Table 4. The R^2^ fitted by Freundlich isothermal model is 0.990. These results suggest that the adsorption of Cr(VI) by bacteria accorded with Freundlich adsorption model in a certain concentration range, indicating that the adsorption of Cr(VI) by bacteria was mainly based on multi-molecular layer adsorption.

**Table 4:** Parameters for Langmuir and Freundlich isotherm equations for the adsorption of Cr(VI) by immobilized biomass.

Langmuir			Freundlich		
a	b	R^2^	K	n	R^2^
2.39	0.73	0.796	1.41	3.22	0.990

### Desorption characteristics of immobilized waste biomass

Results (Table 5) showed that deionized water, alkali solution and salt solution had poor desorption effect, while acid solution had better desorption effect, in particular 1 mol/L hydrochloric acid. The desorption rate was 94.42 ± 0.87% under this condition. This might be due to the fact that H^+^ in acid solution can compete with metal ions for binding sites on the surface of biomass cells, resulting in metal ions desorption. By using desorbents, the immobilized biomass can be recycled, improving efficiency. It is further shown that this method was non-destructive and could be used for multiple adsorption-desorption cycles.

**Table 5:** Desorption of Cr(VI) adsorbed by immobilized biomass.

Desorbent	Desorption rate (%)
Acetic acid (8%)	23.42 ± 0.30
NaOH(1 mol/L)	8.49 ± 0.16
HCl(1 mol/L)	94.42 ± 0.87
HCl (0.5 mol/L)	87.43 ± 0.63
Deionized water	13.46 ± 0.19
NH_4_Cl (0.5 mol/L)	16.43 ± 0.12

## Discussion

With the increase of γ-PGA demand, the production of γ-PGA increased year by year^11^. In the production of γ-PGA, a large number of waste biomass is produced. If it is discharged directly, the environment will be polluted severely. Traditional treatment method of waste biomass is generally used as fertilizer. However, the added value of the treated biomass as fertilizer is relatively low, and there are many similar products and the competition is fierce^2^. By developing it as a biological adsorbent, the added value can be greatly increased.

The comparison of different biosorbents that remove Cr(VI) was shown in Table 6. The order of adsorption rate of different biosorbents was Rossi *et al*. > Sukumar *et al*. > this study > Mona *et al*. > Sivaprakash *et al*. Although Rossi *et al*. reported a high adsorption rate of adsorbents, the following problems still exist: a long adsorption time (180 min), and the adsorbent could not be reused. There were similar problems existing with adsorbent reported by Sukumar *et al*. Regarding the optimum temperature and pH for adsorption, this study differed greatly from that of *B. subtilis* in the literature. The specific reasons might be as follows: (1) different strains of *B. subtilis*; (2) *B. subtili*s used in this study was a strain producing PGA, PGA was a heavy metal absorbent or chelating agent, and residual PGA in waste biomass might change the adsorption characteristics of bacteria. The adsorbent in this study outcompete the other adsorbents in the following ways: (1) The absorbent here has high adsorption rate. Although there was a certain gap between the adsorption rate (99.96% or 98.7%) reported in the literature (Table 6), the gap was not obvious. (2) The absorbent here has a low cost because it can offset the disposal costs of waste biomass from γ-PGA production. (3) The adsorbents in this study could be reused. In the experiment, the adsorbent was reused for 8 times continuously, and it still had good adsorption capacity (data not shown). (4) Short adsorption time (60 min). From Table 6, it could be seen that the adsorption time reported in other literatures was mostly 140–180 min, and the adsorption time in this study was only 60 min, greatly improving the adsorption efficiency. (5) Mild adsorption condition (pH 6.0–7.5, 20–35 °C). From the results of orthogonal experiments, pH and temperature were not significant factors, indicating that within the range of values, the changes of temperature and pH had little effect on the adsorption rate. It was also shown that the waste biomass had better adsorption rate in a wide range of pH (6–7.5) and temperature (20–35 °C). (6) Great economic potential. With the increase of γ-PGA production, a large number of waste biomass is produced. This provides a rich source of raw materials for adsorbent production. The adsorption process reported in this work is simple, has high adsorption efficiency, no secondary pollution, and has good operability. The adsorbent can be further reused, significantly reducing the cos. Therefore, the waste biomass from polyglutamic acid production shown here is an excellent adsorbent with great potentials for application.

**Table 6:** Comparison of different biosorbents in relation Cr(VI).

Biosorbents	Adsorption condition	Adsorption rate (%)	Treating with waste	Reuse	Remarks	Reference
*B. subtilis* SS-1	Initial Cr(VI) concentration (100 mg/L), pH (2), 140 min, 37 °C and biosorbent dose (0.1 g/L).	98.7	No	No	Long adsorption time(140 min); strict adsorption conditions(pH 2, 37 °C)	^12^
*B. subtilis*	Initial Cr(VI) concentration (100 mg/L), pH (2), 140 min, 30 °C and biosorbent dose (2 g/L).	48.64	No	No	Long adsorption time(140 min); strict adsorption condition(pH 2)	^13^
*S. cerevisiae*	Initial Cr(VI) concentration (90 mg/L), pH (5), 180 min, 25 °C and biosorbent dose (5 g/L).	99.66	Yes	No	Long adsorption time(180 min).	^14^
*Cyanobacterial*	Initial Cr(VI) concentration (10 mg/L), pH (4), 180 min, 25 °C and biosorbent dose (0.1 g/L).	90	Yes	No	Long adsorption time(180 min); strict adsorption condition(pH 4)	^15^
*B. subtilis* from γ-PGA production	Initial Cr(VI) concentration (200 mg/L), pH (6.0–7.5), 60 min, 20–35 °C and biosorbent dose (2 g/L).	96.38 ± 0.45	Yes	Yes	Short adsorption time(60 min); mild adsorption condition(pH 6.0–7.5, 20–35 °C); needs of γ-PGA industry	This study

In conclusion, the large amount of waste biomass from the polyglutamic acid industry was used as an adsorbent to adsorb Cr(VI) by trapping it in sodium alginate in an immobilization approach. Orthogonal array design was used to optimize the parameters on biosorption processes of Cr(VI) removal by immobilized waste biomass. The optimal adsorption conditions for immobilized waste biomass were as follows: pH 7.0, initial Cr(VI) concentration of 200 mg/L, 35 °C, biomass of 2 g/L, 60 min. Under these conditions, the absorption efficiency of Cr(VI) was 96.38 ± 0.45%. When the waste biomass was treated with 1 mol/L HCl for 1 h, the desorption rate could reach 94.42 ± 0.87%. It showed that the adsorption accorded with Freundlich adsorption model, indicating that the adsorption of Cr(VI) by bacteria was mainly based on multi-molecular layer adsorption. Physical diffusion and chemical adsorption coexist in the process of adsorption of Cr(VI) by waste biomass, chemical adsorption was dominant. The absorption conditions of waste biomass were mild (pH 6.0–7.5, 20–35 °C) and easily operated. The above results showed that the waste biomass from γ-PGA production was an excellent adsorbent. These investigations would lay a foundation for reducing the pollution of γ-PGA production, exploring a late-model for adsorbent production.

## Materials and Methods

### Preparation of biomass

Waste γ-PGA biomass was collected from Bioengineering Experiment Center of Shandong Jianzhu University, JiNan, China.

Waste biomass was washed twice with distilled water. After centrifugation for 10 min at 4000r/min, the cells were collected, washed twice with l% HCl, washed twice with distilled water, dried at 60 °C, ground into powder after cooling, and then dried for storage.

### Preparation of immobilized bacteria pellets

Sodium alginate(3 g) was put into a flask containing 100 g distilled water and heated in a water bath at 80 °C for about 2 h to form a homogeneous and transparent solution.

The solution was cooled to 40 °C and mixed with 2 g waste biomass powder. The solution was absorbed by syringe and dripped into saturated boric acid solution containing 4% calcium chloride in ice-water bath. The solution was solidified for 24 h, then washed and stored for later use^21,22^.

### Adsorption experiment

Different concentrations of Cr(VI) solutions (50–400 mg/L) were prepared, and pH values of solutions were adjusted to 2–10. Cr(VI) solution (20 mL) was absorbed and transferred into 100 mL flask. Then 3 g waste biomass pellets were put into the flask. In a water-bath oscillator at 10–50 °C and 150 rpm, Cr(VI) was absorbed by waste biomass pellets for 20–200 min. After reaching the reaction time, the sample was taken out and filtered. The residual metal ion concentration in the filtrate was measured by ultraviolet-visible spectrophotometer. The experiments were repeated three times, and the adsorption rate of Cr(VI) by waste biomass was calculated by the concentration of ions in the solution before and after adsorption:

5 $$R/ \% =\frac{\rho 0-\rho 1}{\rho 0}\times 100$$

where R was the adsorption rate (%), *ρ0* was the mass concentration of Cr(VI) in solution before adsorption (mg/L), *ρ1* was the mass concentration of Cr (VI) in solution after adsorption (mg/L).

Adsorption capacity:

6 $${\rm{Q}}({\rm{mg}}/{\rm{g}})=(\rho 0-\rho 1)\times {\rm{v}}/{\rm{m}}$$

where Q was the adsorption capacity (mg/g), *ρ0* was the mass concentration of Cr(VI) in solution before adsorption (mg/L), *ρ1* was the mass concentration of Cr(VI) in solution after adsorption (mg/L), v was the volume of solution(L), m was the dry biomass (g).

### Orthogonal array design

Adsorption was optimized by orthogonal design and L _16_(4^5^) orthogonal table was shown in Table 1^21^. According to the previous single-factor optimization experiments, they were used as the basis for selecting the level of orthogonal design. The five independent factors for adsorption (time, temperature, pH, biomass concentration and initial Cr(VI) concentration) were studied at three different levels and sets of 17 experiments were carried out (Table 1). All experiments were carried out in triplicate. The statistical analysis of the model was represented as an analysis of variance (Table 2).

### Desorption test

After the immobilized biomass adsorbed Cr(VI) in the solution under the optimum adsorption conditions, the yeast was filtered from the adsorption solution. The biomass were equally placed in different desorbents (10 mL), oscillated elution at room temperature for 2 h. The desorption rate was calculated as follows: desorption rate (%) = desorbed Cr(VI) mass/adsorbed Cr(VI) total mass (%).

### Analysis method

The concentration of Cr(VI) was determined according to the method reported in literature^12^.
